# Association of multimorbidity with higher levels of urinary incontinence: a cross-sectional study of 23 089 individuals aged ≥15 years residing in Spain

**DOI:** 10.3399/bjgp20X713921

**Published:** 2020-12-01

**Authors:** Louis Jacob, Guillermo Felipe López-Sánchez, Hans Oh, Jae Il Shin, Igor Grabovac, Pinar Soysal, Petre Cristian Ilie, Nicola Veronese, Ai Koyanagi, Lee Smith

**Affiliations:** Faculty of Medicine, University of Versailles Saint-Quentin-en-Yvelines, Montigny-le-Bretonneux, France.; Faculty of Sport Sciences, University of Murcia, Murcia, Spain.; University of Southern California, Suzanne Dworak Peck School of Social Work, Los Angeles, US.; Department of Pediatrics, Yonsei University College of Medicine, Seoul, Republic of Korea.; Department of Social and Preventive Medicine, Centre for Public Health, Medical University of Vienna, Vienna, Austria.; Department of Geriatric Medicine, Bezmialem Vakif University, Faculty of Medicine, Istanbul, Turkey.; Research and Innovation Department, Queen Elizabeth Hospital, King’s Lynn, UK.; Geriatric Unit, Department of Internal Medicine and Geriatrics, University of Palermo, Palermo, Italy.; ICREA, Barcelona, Spain.; The Cambridge Centre for Sport and Exercise Sciences, Anglia Ruskin University, Cambridge, UK.

**Keywords:** cross-sectional studies, logistic models, multimorbidity, Spain, urinary incontinence

## Abstract

**Background:**

One can assume a relatively high prevalence of urinary incontinence (UI) in people with multimorbidity. However, literature in this area is scarce. There is a need for further robust research to aid GPs to identify patients at a particular risk for UI, and to initiate the early treatment and multidisciplinary management of this condition.

**Aim:**

To examine the association between multimorbidity and UI in 23 089 individuals aged ≥15 years and residing in Spain.

**Design and setting:**

This study used data from the Spanish National Health Survey 2017, a cross-sectional sample of 23 089 participants aged ≥15 years residing in Spain (54.1% female; mean [standard deviation] age = 53.4 [18.9] years).

**Method:**

UI and 30 other physical and mental chronic conditions were self-reported. Multimorbidity was defined as the presence of ≥2 physical and/or mental chronic conditions (excluding UI). Control variables included sex, age, marital status, education, smoking, and alcohol consumption. Multivariable logistic regression analyses were conducted to assess the association between multimorbidity and UI.

**Results:**

The prevalence of UI was 5.9% in this sample. UI was more frequent in the presence than in the absence of each one of the 30 chronic conditions (*P*<0.001). The proportion of people with UI was also higher in the multimorbidity than in the no-multimorbidity group (9.8% versus 0.7%, *P*<0.001). After adjusting for several potential confounders (that is, sex, age, marital status, education, smoking, and alcohol), there was a significant and positive relationship between multimorbidity and UI (odds ratio = 5.02, 95% confidence interval [CI] = 3.89 to 6.59, *P*<0.001).

**Conclusion:**

In this large sample of Spanish individuals aged ≥15 years, suffering from multimorbidity was associated with a significantly higher level of UI.

## INTRODUCTION

According to the International Continence Society, urinary incontinence (UI) is defined as *‘the complaint of any involuntary leakage of urine’*.^1^ The global prevalence of UI is 3–17% in females and 3–11% in males, with varying prevalence across countries.^2^ Although it is most common in older adults, the condition can affect people of all ages. For example, in a study carried out in nulliparous females, the incidence of UI increased from 3% in those aged 25–34 years to 7% in those aged 55–64 years.^3^ Moreover, another study found that the prevalence of moderate–severe UI in community-dwelling females was 7%, 17%, 23%, and 32% in those aged 20–39, 40–49, 60–79, and ≥80 years, respectively.^4^ In studies carried out on males and females, the prevalence of UI was found to be between 11–34% in males aged >65 years, and about twice that frequency in females, proving that UI is more common in females than males.^5^ UI is associated with impaired quality of life^6^ and imposes a tremendous economic burden on national health services/systems.^7^

Multimorbidity is defined as the simultaneous occurrence of ≥2 chronic diseases in one person and can include both physical and mental health complications. It has been found that in developed countries >40% of the population have ≥1 chronic condition, and approximately 25% have >1 condition (that is, multimorbidity).^8^ Similar to UI, multimorbidity is most common among older adults, but can affect people of all ages.^9^ Multimorbidity is a public health concern as it has been shown to be associated with high mortality,^10^ reduced functional status,^11^ and increased use of both inpatient and ambulatory health care.^12^^,^^13^ Multimorbidity is more difficult to manage than singular conditions, and requires close coordination across specialists and generalists.

Owing to a multifactorial aetiology behind UI and that cognitive, neurological, muscular, and urological systems must be robust to maintain continence,^14^^,^^15^ one can assume a relatively high prevalence of UI in people with multimorbidity. However, literature in this area is scarce. In one study of 622 Brazilian females aged ≥50 years it was found that approximately two-thirds of females suffering from UI reported multimorbidity.^16^ The authors of the present study are not aware of any other literature on this topic. Clearly there is a need for further research investigating the association between multimorbidity and UI in population-based samples, among people of multiple ages and from other countries, to establish a better understanding of this topic as well as the underlying risk factors for UI. Consequently, this would help GPs to identify patients at particular risk for UI, and to initiate the early treatment and multidisciplinary management of this condition. Therefore, the aim of the present study was to examine the association between multimorbidity and UI in 23 089 individuals aged ≥15 years residing in Spain.

**Table:** How this fits in

Understanding multimorbidity and urinary incontinence (UI) is critical for medical practitioners. This study found that UI was more frequent in the presence than in the absence of chronic conditions. Those with multimorbidity were five times more likely to suffer from UI. GPs should be aware that those with multimorbidity are at an increased risk of UI.

## METHOD

### The survey

Data from the Spanish National Health Survey 2017 were analysed. This survey was undertaken in Spain between October 2016 and October 2017. Details of the survey method have been already published.^17^ For the data collection, a stratified three-stage sampling was used in which the census sections were first considered, followed by the family dwellings, and then an individual aged ≥15 years was selected within each dwelling. The sections were selected within each stratum with probability proportional to their size. After arrangement by the size of the dwellings, the dwellings in each section were selected with equal probability by systematic sampling. This procedure leads to self-weighting samples in each stratum. For the selection of the person who had to complete the adult questionnaire, the random Kish method was used, which assigns equal probability to all adults in the household. The sample consisted of 23 089 individuals aged 15–103 years residing in Spain. There were no exclusion criteria in the present study, and the overall sample was included in the statistical analyses. The method of data collection used was computer-assisted personal interviewing (CAPI), conducted in the homes of the selected participants. The interviewers, previously trained, completed the questionnaires with the information provided by the participants. All of them signed an informed consent form before responding to the survey questions.

### Multimorbidity (independent variable)

Multimorbidity was defined as the presence of ≥2 chronic conditions (excluding UI). Those who answered affirmatively to the yes/no question ‘Have you ever been diagnosed with *‘*chronic condition’?’ were considered to have the specific chronic condition. All conditions except obesity were assessed with this question. Previous research has confirmed the validity and high accuracy of self-reported diagnosis of chronic conditions.^18^^,^^19^ Using the standard World Health Organization (WHO) definition, obesity was defined as body mass index (BMI) ≥30 kg/m^2^, and BMI <30 kg/m^2^ was considered no obesity.^20^ BMI was calculated as weight in kilograms divided by height in metres squared based on self-reported weight and height. The chronic conditions that were included are listed in Box 1 classified following the International Classification of Diseases, 11th Revision (ICD-11), of the WHO.

**Box 1.** List of chronic conditions classified following the International Classification of Diseases, 11th Revision (ICD-11)^21^

Diseases of the circulatory system	Hypertension Myocardial infarction Angina pectoris and other coronary diseases Other cardiac diseases Varicose veins of lower extremities
Diseases of the nervous system	Stroke Migraine and other frequent headaches
Diseases of the musculoskeletal system or connective tissue	Osteoarthritis Chronic neck pain Chronic low back pain Osteoporosis
Diseases of the immune system	Chronic allergy (excluding allergic asthma)
Diseases of the respiratory system	Asthma (including allergic asthma) Chronic bronchitis, emphysema, or COPD
Diseases of the digestive system	Liver cirrhosis and other hepatic disorders Peptic ulcer disease Chronic constipation Haemorrhoids
Diseases of the genitourinary system	Renal disease
Diseases of the visual system	Cataract
Diseases of the skin	Chronic skin disease
Mental, behavioural, or neurodevelopmental disorders	Depression Anxiety disorder Other psychiatric disorders
Neoplasms	Cancer
Endocrine, nutritional, or metabolic diseases	Thyroid disease Diabetes Hypercholesterolaemia Obesity
Injury, poisoning, or certain other consequences of external causes	Permanent injuries caused by an accident

COPD = chronic obstructive pulmonary disease.

### UI (dependent variable)

Those who answered affirmatively to the question ‘Have you ever been diagnosed with UI?’ were considered to have UI. Previous research has confirmed the validity and high accuracy of self-reported diagnosis of UI.^22^

### Control variables

The selection of the control variables was based on previous studies showing that these factors are associated with both the independent^23^^–^^27^ and the dependent variable.^28^^–^^31^ Sociodemographic variables included sex, age, marital status, and education. Marital status was categorised as married and not married (single/widowed/divorced/separated). Education was based on the highest educational level achieved and was categorised as ≤primary, secondary, and ≥tertiary. Smoking status was self-reported and categorised as never, past, and current smoking. Alcohol consumption in the past 12 months was self-reported and categorised as yes (any) and no (none).

### Statistical analysis

The statistical analysis was performed with R 3.5.2 (the R Foundation, https://www.r-project.org). All the analyses were carried out taking into account the cross-sectional design of the survey, using appropriate tests for this design. Differences in the sample characteristics (by multimorbidity status) and in the prevalence of UI (by chronic condition and multimorbidity status) were assessed by χ^2^ tests for all variables except age (*t*-test). The association between multimorbidity (independent variable) and UI (dependent variable) was assessed using multivariable logistic regression. Independent variables were included in the models as categorical variables with the exception of age, which was included as a continuous variable. UI was included in the model as dichotomous variable. Models were adjusted for basic sociodemographic and behavioral variables (that is, sex, age, marital status, education, smoking, alcohol).^23^^–^^31^ There were missing data for the following variables only: marital status (*n* = 39, 0.17%), smoking (*n* = 22, 0.10%), alcohol consumption (*n* = 26, 0.11%), and obesity (*n* = 1070, 4.63%). Complete-case analysis was carried out. Results from the logistic regression analyses are presented as odds ratios (ORs) and 95% confidence intervals (CIs). Confidence intervals and *P*-values were corrected using the Benjamini–Yekutieli and the Benjamini–Hochberg procedures to control the false discovery rate. The level of statistical significance was set at *P*<0.05.

## RESULTS

**Figure 1.**
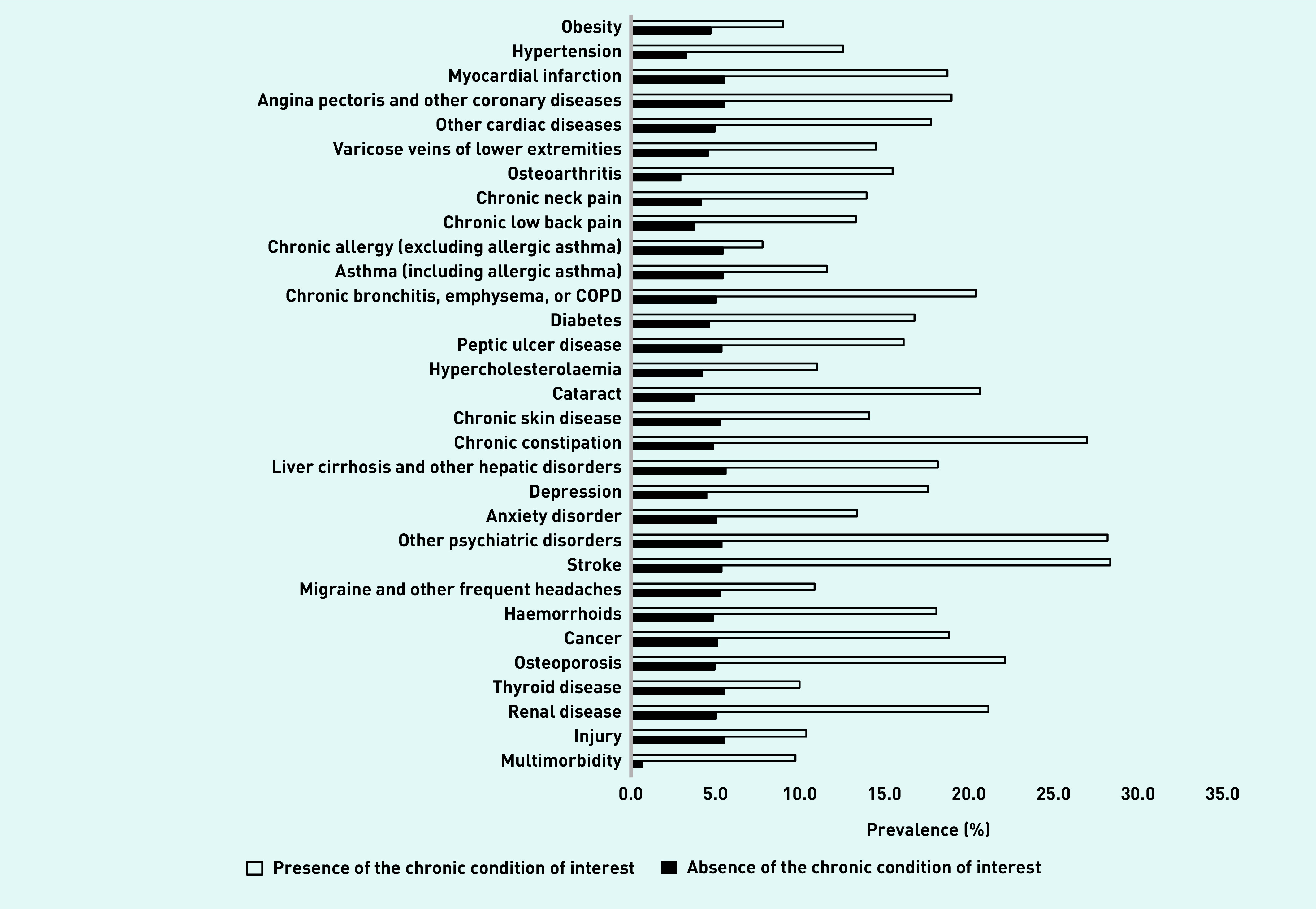
**Prevalence of urinary incontinence by chronic condition and multimorbidity.**^a^ *^a^**Multimorbidity was defined as the presence of two or more chronic conditions. Chronic conditions are listed in Box 1. Urinary incontinence and other chronic conditions except obesity were assessed with questions with ‘yes’ and ‘no’ options. BMI was calculated as weight in kilograms divided by height in meters squared based on self-reported weight and height. Using the standard WHO definition, obesity was defined as BMI ≥30 kg/m^2^, and BMI <30 kg/-m^2^ was considered no obesity. Urinary incontinence was more frequent in the presence than in the absence of each one of the 30 chronic conditions or multimorbidity.*****P *****-values were based on χ^2^ tests and were corrected using the Benjamini–Hochberg adjustment method. All*****P *****-values were lower than 0.001 (not displayed in the figure). BMI = body mass index. COPD = chronic obstructive pulmonary disease. WHO = World Health Organization.***

There were 23 089 individuals aged ≥15 years included in this cross-sectional study (54.1% of females; mean (standard deviation) age = 53.4 (18.9) years; Table 1). The proportion of females, married individuals, people with ≤primary education, past smokers, and people with no alcohol consumption was more frequent in the multimorbidity than in the no-multimorbidity group, whereas people with multimorbidity were older than those without multimorbidity. The prevalence of UI was 5.9% in this sample. UI was more frequent in the presence than in the absence of each one of the 30 chronic conditions (*P*<0.001; Figure 1). The proportion of people with UI was also higher in the multimorbidity than in the no-multimorbidity group (9.8% versus 0.7%, *P*<0.001). The results of the regression analysis are displayed in Table 2. After adjusting for several potential confounders (that is, sex, age, marital status, education, smoking, alcohol), there was a significant and positive relationship between multimorbidity and UI (odds ratio = 5.02, CI = 3.89 to 6.59 *, P*<0.001).

**Table 1.** Sample characteristics (overall and by multimorbidity status)^a^

**Characteristics**	**Category**	**Overall (N = 23 089)**	**Multimorbidity**		***P*-value^b^**

			**No (N = 9940)**	**Yes (N = 13 149)**	
Sex, %, *n*	Male	45.9 (10 595)	51.7 (5141)	41.5 (5454)	<0.001
	Female	54.1 (12 494)	48.3 (4799)	58.5 (7695)	

Age	Mean (SD)	53.4 (18.9)	43.0 (16.0)	61.3 (17.0)	<0.001

Marital status %, *n*	Single/widowed/divorced/separated	45.9 (10 585)	47.4 (4701)	44.8 (5884)	<0.001
	Married	54.1 (12 465)	52.6 (5215)	55.2 (7250)	

Education, %, *n*	≤Primary	31.2 (7206)	15.4 (1532)	43.2 (5674)	<0.001
	Secondary	43.0 (9936)	51.1 (5077)	37.0 (4859)	
	≥Tertiary	25.8 (5947)	33.5 (3331)	19.9 (2616)	

Smoking, %, *n*	Never	50.7 (11 707)	50.4 (5007)	51.0 (6700)	<0.001
	Past	25.8 (5962)	21.3 (2114)	29.3 (3848)	
	Current	23.4 (5398)	28.3 (2807)	19.7 (2591)	

Alcohol, %, *n*	No	35.8 (8260)	28.5 (2834)	41.3 (5426)	<0.001
	Yes	64.1 (14 803)	71.4 (7094)	58.6 (7709)	

a *Multimorbidity was defined as the presence of two or more chronic conditions. Chronic conditions are listed in Box 1. All conditions except obesity were assessed with questions with ‘yes’ and ‘no’ options. BMI was calculated as weight in kilograms divided by height in meters squared based on self-reported weight and height. Using the standard WHO definition, obesity was defined as BMI ≥30 kg/m^2^, and BMI < 30 kg/m^2^ was considered no obesity. Values are % (*N*) unless otherwise stated.*

b P*-values were based on χ^2^ tests except for age (*t*-test), and were corrected using the Benjamini–Hochberg procedure. BMI = body mass index. SD = standard deviation. Tertiary education: known as third-level, third-stage, or post-secondary education. WHO = World Health Organization.*

**Table 2.** Association between multimorbidity (independent variable) and urinary incontinence (dependent variable) in adults residing in Spain^a^

**Characteristics**	**Category**	**Odds ratio**	**95% CI^b^**	***P*-value^c^**
Multimorbidity	No	Reference		
	Yes	5.02	3.89 to 6.59	<0.001

Sex	Male	Reference		
	Female	1.20	1.03 to 1.40	0.020

Age	One-unit increase	1.06	1.05 to 1.06	<0.001

Marital status	Single/widowed/divorced/separated	Reference		
	Married	0.96	0.84 to 1.09	0.498

Education	≤Primary	Reference		
	Secondary	0.74	0.63 to 0.87	<0.001
	≥Tertiary	0.64	0.51 to 0.79	<0.001

Smoking	Never	Reference		
	Past	1.36	1.15 to 1.60	<0.001
	Current	1.13	0.90 to 1.41	0.279

Alcohol	No	Reference		
	Yes	0.73	0.64 to 0.84	<0.001

a Multimorbidity was defined as the presence of two or more chronic conditions. Chronic conditions are listed in Box 1. Urinary incontinence and other chronic conditions except obesity were assessed with questions with ‘yes’ and ‘no’ options. BMI was calculated as weight in kilograms divided by height in meters squared based on self-reported weight and height. Using the standard WHO definition, obesity was defined as BMI ≥30 kg/m^2^, and BMI <30 kg/m^2^ was considered no obesity. Models were adjusted for sex, age, marital status, education, smoking, and alcohol.

b Confidence intervals were corrected using the Benjamini–Yekutieli adjustment method.

c P*-values were corrected using the Benjamini–Hochberg adjustment method. BMI = body mass index. CI = confidence interval. Tertiary education: known as third-level, third-stage, or post-secondary education. WHO = World Health Organization.*

## DISCUSSION

### Summary

In this large sample of the Spanish population, it was found that the prevalence of UI was 5.9%. Moreover, those with multimorbidity were five times more likely to suffer from UI. These findings support previous literature where another study showed in a small sample of Brazilian females that approximately two-thirds of those with UI suffered from multimorbidity.^16^ The present study adds to this literature by showing that such an association exists in a large sample of males and females residing in Spain.

### Strengths and limitations

The present study investigated the relationship between multimorbidity and UI in a large sample of males and females. However, the present findings must be interpreted in light of the study limitations. Both UI and all chronic conditions were self-reported, potentially introducing bias. The stem question asked was ‘Have you ever been diagnosed with “chronic condition”?’ Owing to the wording of the question it is possible that a person used to have a specific chronic condition but no longer does, potentially inflating the prevalence of multimorbidity observed in this study. Furthermore, participants were only asked whether they suffered from UI and not the type of UI; different types of UI may have different associations with multimorbidity, and further research is now required to address this question. Data on duration of chronic conditions were not available, thus potentially introducing some bias into analyses. Moreover, there was no information on parity, although parity is a well known risk factor for UI in females. Data on those who received the invitation to participate in the survey and did not respond are not available. Moreover, there are no recent available national statistics to compare the present sample to. Therefore, the representativeness of the present sample is not known. Finally, the cross-sectional nature of the study means the direction of observed associations is not known. Therefore, future longitudinal studies are needed to clarify the direction of causality. Nevertheless, the mere coexisting presence of UI with chronic conditions and multimorbidity highlights an important health priority and informs targeted intervention.

### Comparison with existing literature

In this large sample of the Spanish population, the prevalence of UI was 5.9% and the prevalence of multimorbidity was 56.9%. This prevalence is comparable with existing literature. For example, a systematic review assessed the global prevalence of UI in different samples of adults from Europe, the United States, Asia, and Africa, obtaining varying UI prevalence estimates with ranges of 1.8–30.5% in European populations, 1.7–36.4% in US populations, and 1.5–15.2% in Asian populations, with prevalence dependent on age and sex.^7^ Regarding the prevalence of multimorbidity, another systematic review including 70 057 611 patients in 12 countries found a multimorbidity prevalence ranging from 12.9–95.1%.^32^

There are several plausible mechanisms that likely increase risk of UI for those suffering from multimorbidity. First, changes in age-related immune functions, hormonal changes, and increasing incidence of comorbid diseases may facilitate urinary tract infections that can result in UI.^33^ Second, poor sleep quality^34^ is associated with nocturia (waking from sleep to void). Third, multimorbidity is associated with mild cognitive impairment^35^^,^^36^ and with cognitive decline^37^ and, in turn, those with a dementia diagnosis (a condition not available to the present study) have approximately three times the rate of diagnosis of UI.^38^ Fourth, another possible mechanism is polypharmacy (often defined as the prescription of ≥5 different drugs), as polypharmacy is strongly associated with multimorbidity,^39^ while the frequency of polypharmacy is high among patients attending a specialist outpatient department for UI.^40^ Fifth, sarcopenia could also have a mediating role in the association between multimorbidity and UI, as previous studies have found associations of sarcopenia with both multimorbidity^41^ and UI.^42^ Sixth, another important factor is physical activity, as less physical activity is associated with a higher prevalence of multimorbidity^43^ and UI.^44^

### Implications for practice

Understanding multimorbidity and UI is critical for medical practitioners. First, in managing multimorbidity, UI can easily be overlooked or eclipsed by other more pressing complaints, especially if patients feel too embarrassed to mention the topic. In this case, it is incumbent upon the medical provider to elicit this information from the patient. These findings suggest that UI should become a standard feature of clinical interviews, given that treatment for UI could involve a number of interventions (for example, pelvic floor exercises, medications, surgery, medical devices) that must figure into already complicated treatment plans for patients with multimorbidity. Second, UI is associated with significant impairment in occupational, social, sexual, and recreational functioning. That is, daily activities may be constrained by geographic proximity to bathrooms. Patients with multimorbidity may already face significant impairment from multiple underlying conditions, and often treatment for these conditions aims to maximise overall functioning and independent living. The presence of UI can interfere with this treatment aim. Third, UI is associated with falls and other injuries that are especially concerning if occurring among people with multimorbidity, given that they are already physically vulnerable, and may likely suffer more serious injuries or take longer to recover. Taking that into account, addressing UI may help prevent further injury.

In conclusion, in this large sample of Spanish individuals aged ≥15 years, suffering from multimorbidity was associated with a significantly higher increased risk of UI. Interventions specifically designed for those with multimorbidity to reduce or manage co-occurring UI are required. Finally, urologists and GPs should be aware that those with multimorbidity are at an increased risk of UI.
